# Effect of a mindfulness program on stress, anxiety, depression, sleep quality, social support, and life satisfaction: a quasi-experimental study in college students

**DOI:** 10.3389/fpsyg.2025.1508934

**Published:** 2025-02-12

**Authors:** Paul Alan Arkin Alvarado-García, Marilú Roxana Soto-Vásquez, Francisco Mercedes Infantes Gomez, Natalia Mavila Guzman Rodriguez, William Gil Castro-Paniagua

**Affiliations:** ^1^Grupo de investigación en Salud Mental y Medicina Integrativa, Escuela de Medicina, Universidad César Vallejo, Trujillo, Peru; ^2^Universidad Nacional de Trujillo, Trujillo, Peru; ^3^Escuela de Psicología, Universidad César Vallejo, Trujillo, Peru; ^4^Universidad Nacional José María Arguedas, Andahuaylas, Peru

**Keywords:** mindfulness, stress, anxiety, depression, sleep quality, social support, life satisfaction, college students

## Abstract

**Introduction:**

The university experience often brings various personal and academic challenges that can negatively impact students’ mental health. This research aimed to evaluate the effect of a mindfulness program on stress, anxiety, depression, sleep quality, social support, and life satisfaction among university students.

**Methods:**

A quasi-experimental study was conducted with 128 participants, divided into experimental and waiting list control groups. The experimental group participated in a mindfulness meditation program consisting of 12 weekly sessions. Pre-test and post-test measurements were performed using the Perceived Stress Scale (PSS-10), the Zung Self-Rating Anxiety Scale (SAS), the Zung Self-Rating Depression Scale (SDS), the Pittsburgh Sleep Quality Index (PSQI), the Medical Outcomes Study Social Support Survey (MOS-SS), and the Satisfaction with Life Scale (SWLS) to assess the variables.

**Results:**

The experimental group showed statistically significant differences between the study phases and the groups after the intervention for all the variables examined (*p* < 0.05). The effect sizes calculated using the HC3 model were stress (*η*^2^ = 0.376), anxiety (*η*^2^ = 0.538), depression (*η*^2^ = 0.091), sleep quality (*η*^2^ = 0.306), social support (*η*^2^ = 0.704), and life satisfaction (*η*^2^ = 0.510). The mindfulness program was shown to be effective in reducing levels of stress, anxiety, and depression while also improving sleep quality, social support, and life satisfaction in college students.

**Conclusion:**

These findings indicate that mindfulness meditation may be valuable for enhancing psychological well-being in educational settings.

## 1 Introduction

Mental health challenges, including stress, anxiety, and depression, are increasingly prevalent among college students, with significant implications for their academic performance and overall well-being (Wu and Liu, 2024). These concerns are particularly pressing in lower-middle-income countries and among students pursuing health sciences careers, where the convergence of academic, clinical, and social pressures compounds mental health risks (Li et al., 2022; Agyapong-Opoku et al., 2024). As students transition to college, they often face a host of novel stressors—greater academic rigor, new social environments, and increased autonomy—that can trigger or exacerbate mental health conditions if not effectively managed (Felton et al., 2022). Unaddressed psychological distress during these formative years may serve as a precursor to long-term health problems, underscoring the need for timely and effective interventions (Emmerton et al., 2024; Turner and Holdsworth, 2024).

In addition to stress, anxiety, and depression, sleep quality has emerged as a critical yet frequently overlooked aspect of student mental health. Inadequate or fragmented sleep patterns can heighten emotional reactivity, impair cognitive function, and lead to a vicious cycle in which stress and worry further disrupt sleep (Perkinson-Gloor et al., 2013; Sun et al., 2024). Over time, poor sleep quality has been linked to increased symptoms of depression and anxiety and a decline in academic performance (Becker et al., 2018). Another essential factor is social support, the perceived availability of emotional, instrumental, or informational aid from peers, family, or community members (Semmer et al., 2008). Strong social networks have been shown to buffer the negative effects of stress and loneliness, improving overall well-being (Franco-O’Byrne et al., 2023). Conversely, insufficient social support may exacerbate psychological distress and hinder students’ ability to cope with academic and personal challenges (Vungkhanching et al., 2017; Dadandı and Çıtak, 2023).

Life satisfaction, or a person’s overarching evaluation of their quality of life, is closely interlinked with these mental health factors (Bramhankar et al., 2023). High levels of stress, anxiety, and depression often diminish life satisfaction, but strong social support and efficient coping mechanisms enhance it (Chia et al., 2024; Çiçek et al., 2024). Given the multitude of pressures university students confront, there is a growing imperative to develop interventions that address these intersecting domains—stress, anxiety, depression, sleep quality, social support, and life satisfaction—comprehensively.

Mindfulness, rooted in ancient meditative practices, has gained considerable attention as a widely accepted complementary and alternative medicine (CAM) approach for improving mental health outcomes among college students (Simonsson et al., 2021; Zheng and Yang, 2024). Defined as the nonjudgmental awareness and acceptance of the present moment, mindfulness cultivates a compassionate orientation toward one’s experiences, fostering emotional regulation and resilience (Bishop et al., 2004; Kabat-Zinn, 2005).

The theoretical basis for mindfulness lies in its capacity to enhance metacognitive awareness, reduce cognitive reactivity, and regulate the hypothalamic–pituitary–adrenal (HPA) axis, reducing cortisol release and thereby alleviating the physiological and psychological symptoms associated with stress and anxiety (Garland et al., 2015). Furthermore, the theoretical framework for mindfulness includes its ability to enhance interoceptive awareness—an individual’s ability to perceive internal bodily states (Farb et al., 2015). Enhanced interoceptive awareness strengthens the experiential self (moment-to-moment awareness). It reduces overidentification with the narrative self (the self-concept over time), promoting a sense of presence and emotional stability (Gibson, 2019).

Previous literature has shown that mindfulness-based interventions can improve psychological well-being in university students, decreasing symptoms of depression, anxiety, stress, and insomnia (Hall et al., 2018; Gallo et al., 2023; Zhang, 2024). However, a systematic review showed that evidence on sleep quality is inconclusive. Although scientific evidence highlights mindfulness’s great potential for the mental health of university students, further research is needed to clearly understand its effects and how they would work in an academic environment (Dawson et al., 2020; Zuo et al., 2023). Evidence also links mindfulness to enhanced social support and life satisfaction, as the practice can increase empathy, emotional regulation, and interpersonal effectiveness (Wilson et al., 2020; MacDonald et al., 2024). Moreover, much of the current research is derived from Western contexts, raising questions about cultural variability in the acceptance and effectiveness of mindfulness-based interventions. It is essential to explore the effectiveness of these interventions in different cultural settings to develop more inclusive and effective programs.

This study aims to address these gaps by evaluating the effects of a mindfulness meditation program on stress, anxiety, depression, sleep quality, social support, and life satisfaction among college students in a culturally diverse academic setting. By comprehensively analyzing these variables, this research seeks to expand the understanding of mindfulness-based interventions and their adaptability across cultural contexts. Ultimately, these insights aim to inform the development of inclusive and effective mental health programs for college students worldwide, contributing to a holistic approach to mental health in higher education.

## 2 Materials and methods

### 2.1 Study design, sample, and ethics

The study employed a quasi-experimental research design with pre-and post-test evaluations. A power analysis was completed using ‘G Power 3’ with a moderate effect size, an *α* level of 0.05, and a power of 0.80. The number of participants required to determine the difference in effect was 128, 64 per group, comprising a control group (CG), which served as a waiting list without treatment, and the experimental group (GE), whose participants underwent a 12-session mindfulness meditation program based on a previous study (Alvarado-García et al., 2022a). The School of Medicine Research Ethics Committee of Cesar Vallejo University, Trujillo, Perú, approved the study protocol (Approval number: 021-CEI-EPM-UCV-2023 - 26/04/2023).

### 2.2 Instruments

#### 2.2.1 Perceived stress scale (PSS-10)

This 10-item scale measures stressful life circumstances and situations. Respondents are asked to indicate their frequency of occurrence on a 5-point Likert scale (never = 0; almost never = 1; sometimes = 2; fairly often = 3; very often = 4). Items 4, 5, 7, and 8 were reversed (Cohen, 1988). For this study, the validity and reliability test for the local population and context was determined using the item test method, with values greater than 0.48 for each item; additionally, the reliability coefficient of 0.98 was found using the split-half method.

#### 2.2.2 Zung self-rating anxiety scale (SAS)

This scale consists of 20 items, scored from 1 to 4 (1 = none or a little of the time, 2 = some of the time, 3 = good part of the time, 4 = most of the time). The validity and reliability coefficients for the local university population were determined in a previous study, where coefficients greater than 0.40 were found using the item-test method, and a coefficient of 0.89 was found using the split-half method (Alvarado-García et al., 2022b).

#### 2.2.3 Zung self-rating depression scale (SDS)

This scale consists of 20 items. Each item is scored from 1 to 4 (1 = none or a little of the time, 2 = some of the time, 3 = good part of the time, 4 = most of the time). The validity and reliability coefficients for the local university population were determined in a prior study. Coefficients above 0.30 were found by the item-test method, and a coefficient of 0.94 was found using the split-half method (Alvarado-García et al., 2022b).

#### 2.2.4 Pittsburgh sleep quality inventory (PSQI)

This questionnaire contains 18 items grouped into seven components. The score of each item ranges from 0 to 3. The sum of these seven components is the total PSQI score, 0–21. The validity and reliability coefficients for the local university population were determined in a prior study. Coefficients greater than 0.42 for each item were found using the item-test method, and a coefficient of 0.96 was found using the split-half method (Alvarado-García et al., 2023b).

#### 2.2.5 The medical outcomes/ study social support survey (MOS-SS)

This questionnaire consisted of 20 items. The first item reported on the size of the social network. The subsequent 19 items are rated on a five-point Likert-type scale that ranged from 1 “never” to 5 “always” (Sherbourne and Stewart, 1991). For this study, the validity and reliability test for the local university population was determined in a pilot test using the item test method, with values greater than 0.43 for each item; additionally, a reliability coefficient of 0.96 was found using the split-half method.

#### 2.2.6 Satisfaction with life scale (SWLS)

This is a brief 5-item scale designed to assess global cognitive scores of life satisfaction. Participants responded on a 7-point Likert scale, where higher scores indicate greater satisfaction with life (Diener et al., 1985). For this study, the validity and reliability test for the local university population was determined in a pilot test using the item test method, with values greater than 0.51 for each item; additionally, a reliability coefficient of 0.94 was found using the split-half method.

### 2.3 Study procedure

**Figure 1:**
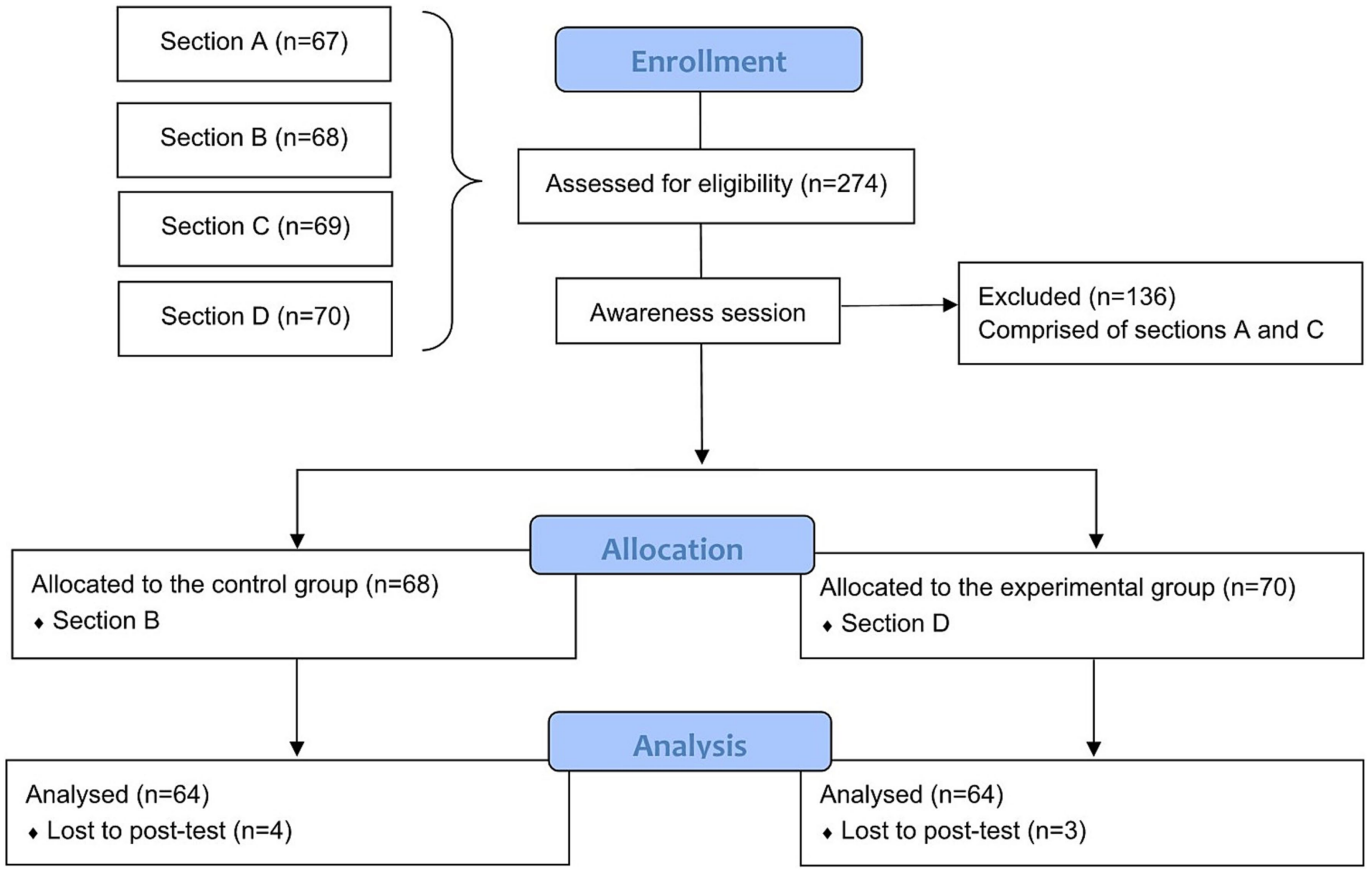
Flowchart of the study. This figure illustrates the study’s enrollment, allocation, intervention, and analysis phases, adapted from CONSORT guidelines.

The present study involved health sciences students enrolled at a private institution in Peru. An awareness session was conducted in all four sections (A, B, C, and D) of a research course. This session aimed to introduce the concept of mindfulness. The primary objective was to inform students and foster an understanding of the intervention’s relevance and benefits for mental health. Following the awareness session, students completed an interest survey with the question, “If given the opportunity to participate in a mindfulness program, would you be willing to join?” This survey gaged willingness to participate rather than preferences for the program. While the responses were slightly higher in sections B and D, the differences were not statistically significant (Supplementary Table S2). These sections were selected to ensure sufficient participation and adherence to the program. Section B was designated as the control group (CG), and Section D was assigned as the experimental group (EG) (Figure 1, Flowchart of the study). The study included university students currently enrolled in a research course. Participants were required to meet specific scores on the instruments: PSS-10 scores >9, SAS and SDS scores >49, and PSQI scores >5. These thresholds were selected to ensure the inclusion of individuals who might benefit the most from the mindfulness program. Additionally, all participants provided written informed consent and committed to attending all mindfulness sessions and completing pre-and post-test assessments. On the other hand, participants were excluded if they had prior experience with mindfulness-related practices such as meditation, tai chi, or yoga to avoid potential bias from pre-existing familiarity with these techniques. Those who underwent psychiatric treatment, took psychotropic medications, or actively experienced substance abuse were also excluded to minimize confounding variables. Pregnant individuals were not eligible to participate, given the physiological and psychological changes associated with pregnancy that could influence the study variables. Furthermore, participants enrolled in other psychological or wellness interventions during the study period were excluded to ensure the integrity of the study’s findings. After the groups were established, a pretest was administered with all the instruments. Then, 12 weekly sessions were booked, each lasting 60 min. The mindfulness meditation program was run under the direction of a qualified mindfulness teacher. There were also compliance report forms and daily mindfulness meditation audio recordings. After completing the program, the post-test was conducted in both groups using the same methodology as the initial evaluation. The duration of the program lasted 3 months. Furthermore, all participants received detailed information on the research goals. They indicated their agreement to participate by signing an informed consent form distributed alongside the pretest. This process ensured that the identities of the participants remained anonymous. It was also specified that the collected data would be treated with the utmost confidentiality. The research followed the ethical principles of the Declaration of Helsinki (World Medical Association, 2013).

### 2.4 Data analysis

The means and standard deviation (SD) of all variables were determined. Pearson’s Chi-square and Fisher’s exact tests examined differences in the participants’ sociodemographic and clinical data. The Wilcoxon and Mann–Whitney *U* tests assessed differences between study phases and groups. These tests were selected because the data did not conform to a normal distribution. In addition, covariance (ANCOVA) analysis was performed to control for baseline differences, with pre-test scores included as covariates. Considering deviations from normality and homoscedasticity, the robust HC3 model was applied to provide more accurate standard errors. Mediation and moderation analyses were conducted using the PROCESS macro (models 4 and 1, respectively) with 5,000 bootstrap samples. All analyses were performed using Prism 8 (GraphPad, CA, USA), SPSS version 27 (IBM Corp., Armonk, NY, USA), and PROCESS version 4.2.

## 3 Results

### 3.1 Sociodemographic and clinical characteristic

Table 1 displays the socio-demographic and clinical data of the analyzed participants, comprising 51 (39.8%) males and 77 (60.2%) females. The CG group comprised 27 males (42.2%) and 37 females (57.8%). In contrast, the EG group comprised 24 males (37.5%) and 40 females (62.5%). Regarding age, the range from 18 to 25 and 26 to 35 was the majority, with 52 (40.6%) and 69 (53.9%) participants with almost the same distribution in both groups. Gender and age showed no statistically significant differences (*p* > 0.05). Most participants of CG and EG are unmarried in terms of marital status. Likewise, the majority of both groups did not have any clinical treatment. There were no statistically significant differences between the groups regarding marital status and clinical treatment provided (*p* > 0.05).

**Table 1:** Participant socio-demographic and clinical data.

Socio-demographic data	CG	EG	Total	*p*-value
Gender				
Male	27 (42.2%)	24 (37.5%)	51 (39.8%)	0.588^a^
Female	37 (57.8%)	40 (62.5%)	77 (60.2%)	
Age (yr)				
18–25	28 (43.8%)	24 (37.5%)	52| (40.6%)	0.666^b^
26–35	32 (50.0%)	37 (57.8%)	69 (53.9%)	
36–45	4 (6.2%)	3 (4.7%)	7 (5.5%)	
Marital status				
Married	2 (3.1%)	1 (1.6%)	3 (2.3%)	0.555^b^
Unmarried	62 (96.9%)	63 (98.4%)	125 (97.7%)	
Clinical treatment provided				
Psychological	7 (10.9%)	5 (7.8%)	12 (9.4%)	0.544^a^
Pharmacological	0 (0%)	0 (0%)	0 (0%)	
None	57 (89.1%)	59 (92.2%)	116 (90.6%)	

a *p-value is calculated by the Pearson Chi-Square test.*

b *p-value is calculated by the Likelihood ratio test.*

### 3.2 Effect of the mindfulness intervention on stress

**Figure 2:**
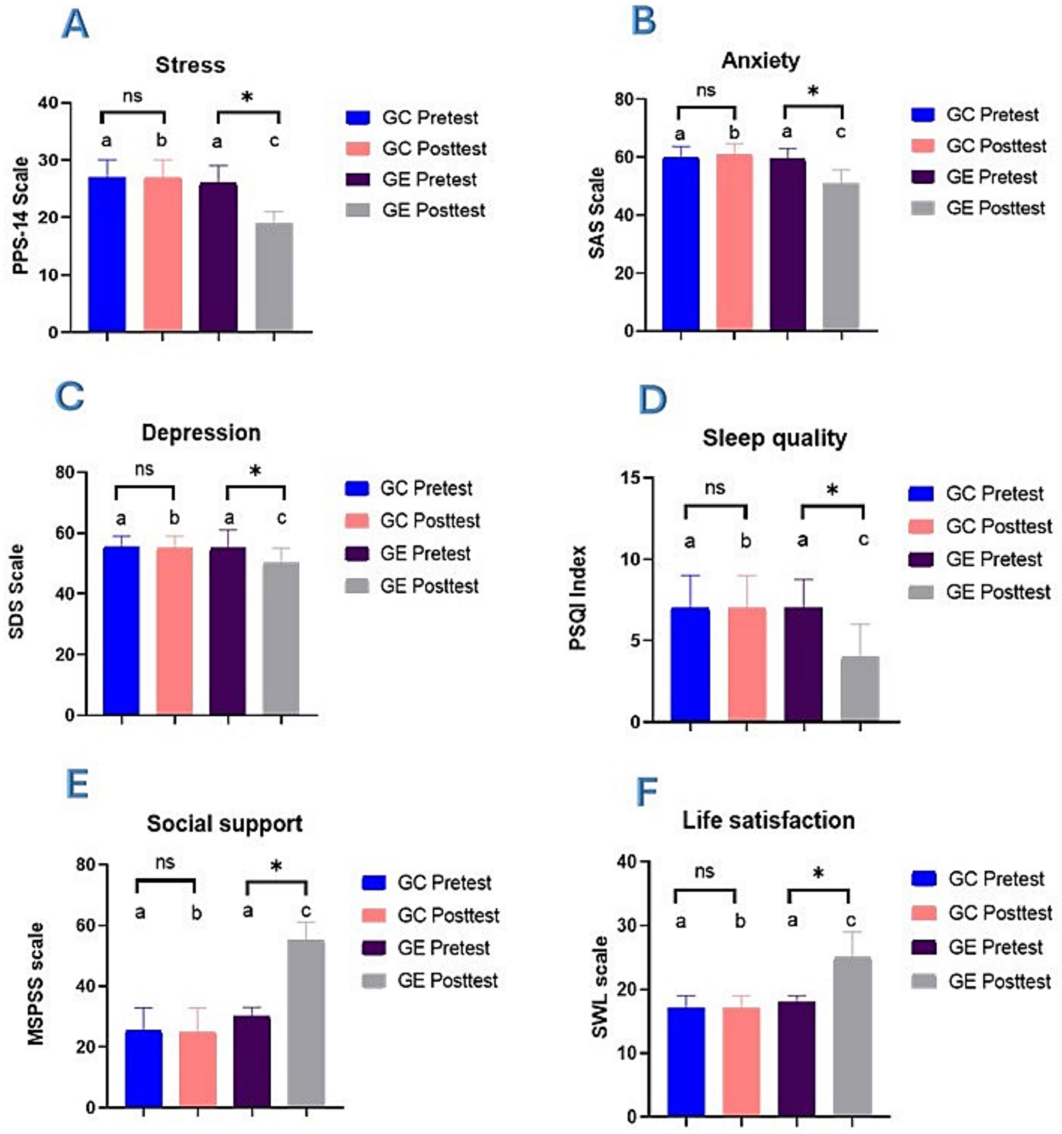
Changes in psychological variables before and after mindfulness intervention. It represents the median and interquartile range (IQR) for **(A)** Stress, **(B)** Anxiety, **(C)** Depression, **(D)** Sleep quality, **(E)** Social support, and **(F)** Life satisfaction. Bars sharing the same lowercase letter do not differ significantly, while bars with different letters denote significant differences between groups (*p* < 0.05) as determined by the Mann–Whitney *U* test. The ‘ns’ symbol denotes no significant difference between pre-and post-intervention phases within the same group, while the asterisk (*) indicates a significant difference (*p* < 0.05) within phases based on the Wilcoxon test.

Figure 2A, displays changes in stress scores. Medians and IQR were used since the data did not conform to normal distribution. Regarding stress (A), there was no significant difference between CG and EG at the pretest (*p* = 0.770). However, in the posttest, significant differences were shown (*p* = 0.000) according to the Mann–Whitney U test. In addition, when comparing study phases, the CG showed no significant differences between the pretest and posttest phases (*p* = 0.159), remaining constant in both phases. In contrast, in the EG, the stress level significantly decreased after the intervention (*p* = 0.000), with the median reducing from 26.00 (IQR: 22.75–29.00) in the pretest to 19.00 (IQR: 15.00–21.00) in the posttest, showing significant differences (*p* < 0.05), according to Wilcoxon test.

### 3.3 Effect of the mindfulness intervention on anxiety

Figure 2B, illustrates the changes in anxiety scores, where CG and EG did not show significant differences at the pretest (*p* = 0.716). However, in the post-test phase, significant differences were observed between both groups (*p* = 0.000). When comparing the study phases, the CG did not show significant differences between the pretest and posttest (*p* = 0.061), remaining stable. In contrast, the EG presented a significant reduction in anxiety after the intervention (*p* = 0.000), with a decrease in the median from 59.50 (IQR: 57.25–63.00) in the pretest to 51.00 (IQR: 48.00–55.75) in the posttest, showing statistical differences (*p* < 0.05).

### 3.4 Effect of the mindfulness intervention on depression

Depression outcomes are displayed in Figure 2C, where no significant differences were found between groups in the pretest phase (*p* = 0.694). However, in the post-test phase, significant differences were detected between both groups (*p* = 0.000). Regarding the study phases, the CG did not show significant changes between the pretest and posttest (*p* = 0.985). At the same time, the EG experienced a significant reduction in depression levels after the intervention (*p* = 0.000), with a median that went from 55.00 (IQR: 50.00–61.00) in the pretest to 50.00 (IQR: 48.00–55.00) in the posttest (*p* < 0.05).

### 3.5 Effect of the mindfulness intervention on sleep quality

Figure 2D, illustrates the changes in sleep quality scores. There were no significant differences between the CG and the EG (*p* = 0.825) in the pretest; however, in the posttest phase, significant differences were observed between the groups (*p* = 0.000). When comparing the study phases, the CG showed no significant differences between the pretest and posttest (*p* = 0.062). In contrast, in the EG, a significant improvement in sleep quality was observed after the intervention (*p* = 0.000), with the median decreasing from 7.00 (IQR: 4.00–8.75) to 4.00 (IQR: 2.00–6.00).

### 3.6 Effect of the mindfulness intervention on social support

Figure 2E, shows the changes in social support scores. At the pretest, no significant differences were observed between the CG and EG (*p* = 0.201); but the post-test groups showed significant differences (*p* = 0.000). Regarding the study phases, an effect of mindfulness was demonstrated in the posttest phase, where a significant increase in social support was observed after the intervention (*p* = 0.000) in the EG, with an increase in the median from 30.00 (IQR: 21.25–33.00) in the pretest to 55.00 (IQR: 46.00–61.00) in the posttest.

### 3.7 Effect of the mindfulness intervention on life satisfaction

Figure 2F, depicts the changes in life satisfaction scores. At the pretest, no significant differences were observed between the CG and the EG (*p* = 0.810). In contrast, in the post-test phase, significant differences were observed between the groups (*p* = 0.000). There were no significant changes in the CG between the pretest and posttest phases (*p* = 0.073). In contrast, in the EG, life satisfaction increased significantly after the intervention (*p* = 0.000), with the median increasing from 18.00 (IQR: 14.25–19.00) in the pretest to 25.00 (IQR: 22.00–29.00) in the posttest.

In addition, Supplementary Table S1 provides detailed descriptive and inferential statistics for stress, anxiety, depression, sleep quality, social support, and life satisfaction. This table summarizes median scores, IQR, and statistical comparisons between the CG and the EG across the pretest and posttest phases. These results support the findings in Figure 2 and provide a comprehensive overview of the observed changes in both groups.

To further validate these findings, ANCOVA and HC3 models were performed to assess the robustness of the intervention effects on each variable, adjusting for baseline differences and ensuring the reliability of the observed outcomes.

### 3.8 ANCOVA and robust HC3 analysis results

ANCOVA was conducted to confirm these findings while accounting for potential covariates (Table 2). As the data did not follow a normal distribution and to account for potential heteroscedasticity, parameter estimates with robust standard errors were also computed using the HC3 method (Table 3). In this sense, Table 2 demonstrates that the mindfulness intervention had a significant effect on post-intervention stress levels, as evidenced by the model’s F-statistic (47.209, *p* < 0.001) and the large effect size (partial *η*^2^ = 0.430). Moreover, the group effect (*F* = 76.940, *p* < 0.001, *η*^2^ = 0.381) confirms that the experimental group experienced a considerable reduction in stress after the intervention. In addition, the statistical power for these effects was 1.000, indicating high confidence in the results. The HC3 robust model further confirms these findings in Table 3, where the group effect remains significant (*B* = 8.090, *p* < 0.001, partial *η*^2^ = 0.376), with an observed power of 1.000, ensuring the robustness of the intervention’s effects. Similarly, the ANCOVA analysis for anxiety reveals a significant reduction in anxiety levels post-intervention, with the model showing a large effect size (partial *η*^2^ = 0.586) and a significant group effect (*F* = 150.647, *p* < 0.001, *η*^2^ = 0.547). These results were also supported by the robust standard error model, where the group effect remained significant (*B* = 9.800, *p* < 0.001, partial *η*^2^ = 0.538). This highlights the intervention’s effectiveness in reducing anxiety levels.

**Table 2:** ANCOVA results for psychological variable.

Dependent variable	Source	Sum of squares	df	Mean square	F	*p*-value	H	Observed power
Post-stress	Model	2570.312	2	1285.156	47.209	<0.001	0.430	1.000
	Pre-stress	465.929	1	465.929	17.115	<0.001	0.120	0.984
	Groups	2094.545	1	2094.545	76.940	<0.001	0.381	1.000
Post-anxiety	Model	3603.862	2	1801.931	88.414	<0.001	0.586	1.000
	Pre-anxiety	453.667	1	453.667	22.260	<0.001	0.151	0.997
	Groups	3070.258	1	3070.258	150.647	<0.001	0.547	1.000
Post-depression	Model	417.702	2	208.851	6.340	0.002	0.092	0.893
	Pre-depression	0.819	1	0.819	0.025	0.875	0.000	0.053
	Groups	417.700	1	417.700	12.679	<0.001	0.092	0.942
Post-sleep quality	Model	869.743	2	434.871	93.847	<0.001	0.600	1.000
	Pre-sleep quality	638.618	1	638.618	137.817	<0.001	0.524	1.000
	Groups	243.745	1	243.745	52.601	<0.001	0.296	1.000
Post-social support	Model	25019.104	2	12509.552	191.140	<0.001	0.754	1.000
	Pre-social support	3749.573	1	3749.573	57.292	<0.001	0.314	1.000
	Groups	20476.060	1	20476.060	312.865	<0.001	0.715	1.000
Post-life satisfaction	Model	2,634,929	2	1317.464	84.462	<0.001	0.575	1.000
	Pre-social support	697.397	1	697.397	44.710	<0.001	0.263	1.000
	Groups	2065.615	1	2065.615	132.425	<0.001	0.514	1.000

**Table 3:** Parameter estimates with robust standard errors.

Dependent variable	Parameter	B	Robust Std. error	*t*	*p*-value	95% Confidence Interval	Partial *η*2	Observed power
Post-stress	Intercept	8.077	2.538	3.183	0.002	[3.055–13.100]	0.075	0.885
	Pre-stress	0.403	0.100	4.026	<0.001	[0.205–0.601]	0.115	0.979
	Group [1 vs. 2]	8.090	0.933	8.675	<0.001	[6.245–9.936]	0.376	1.000
Post-anxiety	Intercept	23.784	5.990	3.971	<0.001	[11.929–35.638]	0.112	0.976
	Pre-anxiety	0.458	0.101	4.551	<0.001	[0.259–0.657]	0.142	0.995
	Group [1 vs. 2]	9.800	0.813	12.058	<0.001	[8.192–11.409]	0.538	1.000
Post-depression	Intercept	51.120	3.867	13.218	<0.001	[43.466–58.774]	0.583	1.000
	Pre-depression	0.013	0.070	0.181	0.856	[−0.126–0.151]	0.000	0.054
	Group [1 vs. 2]	3.616	1.019	3.548	<0.001	[1.599–5.633]	0.091	0.941
Post-sleep quality	Intercept	0.265	0.536	0.495	0,621	[−0.795–1.326]	0.002	0.078
	Pre-sleep quality	0.582	0.078	7.420	<0.001	[0.427–0.737]	0.306	1.000
	Group [1 vs. 2]	2.760	0.390	7.079	<0.001	[1.989–3.532]	0.286	1.000
Post-social support	Intercept	34.951	3.152	11.089	<0.001	[28.713–41.188]	0.496	1.000
	Pre-social support	0.643	0.094	6.875	<0.001	[0.458–0.829]	0.274	1.000
	Group [1 vs. 2]	−25.319	1.470	−17.225	<0.001	[−28.228- −22.410]	0.704	1.000
Post-life satisfaction	Intercept	13.244	1.909	6.939	<0.001	[9.467–17.021]	0.278	1.000
	Pre-life satisfaction	0.681	0.101	6.721	<0.001	[0.481–0.882]	0.265	1.000
	Group [1 vs. 2]	−8.047	0.705	−11.412	<0.001	[−9.443- −6.652]	0.510	1.000

In addition, the ANCOVA analysis for depression revealed a significant effect with an *F*-value of 6.340, *p* = 0.002, and a moderate effect size (partial *η*^2^ = 0.092) with an observed power of 0.893. The HC3 model, which adjusts for heteroscedasticity, confirmed this effect, with the group difference remaining significant (*p* < 0.001) and a confidence interval that does not cross zero [1.599–5.633]. The observed power was 0.941, further reinforcing the robustness of these findings. Although the partial *η*^2^ value of 0.091 indicates a modest effect, the significant results in both the ANCOVA and HC3 models suggest that the mindfulness intervention had a meaningful impact on reducing depression levels in the experimental group. For sleep quality, both models show consistent findings. The ANCOVA results (Table 2) show a significant effect of the intervention (*F* = 52.601, *p* < 0.001, partial *η*^2^ = 0.296), with an observed power of 1.000. The HC3 model confirms these findings, with a significant group effect (*B* = 2.760, *p* < 0.001, partial *η*^2^ = 0.286) and an observed power of 1.000, demonstrating the consistent impact of mindfulness on enhancing sleep quality. In addition, social support showed the most substantial effect of the intervention, as seen in both the ANCOVA (*F* = 312.865, *p* < 0.001, partial *η*^2^ = 0.715) and the robust model (*B* = −25.319, *p* < 0.001, partial *η*^2^ = 0.704), indicating that mindfulness had a profound influence on improving participants perceived social support. These results suggest that the intervention was particularly effective in fostering a sense of social connectedness. Finally, life satisfaction also improved significantly following the intervention. The ANCOVA analysis showed a significant group effect (*F* = 132.425, *p* < 0.001, partial *η*^2^ = 0.514), which was mirrored by the robust model results (*B* = −8.047, *p* < 0.001, partial *η*^2^ = 0.510). These findings indicate that mindfulness contributed to enhancing life satisfaction among the participants.

### 3.9 Exploration of mediators and moderators

As the results of the ANCOVA and HC3 models revealed significant effects on sleep quality, social support, and life satisfaction, we explored their potential as mediators or moderators in explaining the reduction in stress, anxiety, and possibly depression after the mindfulness intervention. In this sense, Table 4 shows the mediation analysis, where the direct effect of the mindfulness intervention on anxiety, as mediated by sleep quality, was negative (−0.30), but the indirect effect had a *p*-value of 0.07, indicating no significance. For stress, social support was tested as a mediator. Although the direct effect was also negative (−0.40), the indirect effect showed a borderline significance (*p* = 0.05). While this suggests a potential partial mediation, it is not strong enough to be considered a definitive mediating effect; however, further research is needed to confirm this role. For life satisfaction as a mediator of depression, neither the direct (−0.20) nor the indirect effect (−0.01) was significant (*p* = 0.15). Finally, Table 5 shows the moderation analysis where the interaction terms between the group and the moderators were all non-significant. Specifically, social support did not significantly moderate the relationship between mindfulness and anxiety (*p* = 0.45), and neither life satisfaction nor sleep quality moderated the effects on stress (*p* = 0.65) or depression (*p* = 0.75), respectively. These results suggest that although social support and life satisfaction play key roles in the psychological outcomes post-intervention, they do not act as significant moderators in this context.

**Table 4:** Mediation analysis results for the effect of mindfulness on psychological outcomes.

X (Predictor)	M (Mediator)	Y (Outcome)	Direct effect (B)	Indirect effect (B)	Total effect (B)	95% CI (Indirect)	*p*-value (Indirect)
Group	Sleep quality	Anxiety	−0.3	−0.05	−0.35	[−0.100, 0.000]	0.07
Group	Social support	Stress	−0.4	−0.08	−0.48	[−0.150, 0.000]	0.05
Group	Life satisfaction	Depression	−0.2	−0.01	−0.21	[−0.050, 0.030]	0.15

**Table 5:** Moderation analysis results for the effect of mindfulness on psychological outcomes.

X (Predictor)	W (Moderator)	Y (Outcome)	Main effect of X	Main effect of W	Interaction (X*W)	95% CI (Interaction)	*p*-value (Interaction)
Group	Social support	Anxiety	−0.3	−0.1	0.05	[−0.200, 0.300]	0.45
Group	Life satisfaction	Stress	−0.4	−0.05	0.02	[−0.150, 0.200]	0.65
Group	Sleep quality	Depression	−0.2	−0.05	−0.01	[−0.100, 0.080]	0.75

## 4 Discussion

Students’ mental health is essential in helping them develop their potential. A positive mental health status improves students’ ability to concentrate, manage stress, and adapt to academic challenges, crucial skills for academic success (Garces et al., 2024). Mindfulness benefits college students by improving mental health, developing coping skills, and promoting adjustment to the educational environment (Ramler et al., 2016).

Several studies agree with our results, revealing that mindfulness interventions reduce symptoms of anxiety, depression, stress, and insomnia (Sousa et al., 2021; Gallo et al., 2023; González-Martín et al., 2023). The psychological and neurological mechanisms that underlie mindfulness act synergistically. Mindfulness enables individuals to observe their thoughts and emotions non-judgmentally. This practice mitigates the activation of the hypothalamic–pituitary–adrenal axis (HPA) axis responsible for stress responses, thus reducing cortisol levels (Bergen-Cico et al., 2014; González-Martín et al., 2023). At the same time, this practice improves emotional regulation of the prefrontal cortex—key in modulating decision-making and emotional responses—and decreases amygdala activity, reducing fear and anxiety (Ng et al., 2023; Zheng et al., 2024). This dual regulation not only alleviates symptoms of anxiety and depression but also enhances sleep quality by reducing mental hyperactivation, a significant barrier to rest.

Furthermore, by reducing rumination, mindfulness interrupts the cycles of repetitive negative thoughts characteristic of depression, fostering emotional well-being (Sommerhoff et al., 2023). This would act as a holistic mechanism whose cascading effect would not only alleviate depressive symptoms but would also reduce anxiety by decreasing constant worry and stress by improving the ability to face difficult situations without an exaggerated emotional reaction. Likewise, greater emotional well-being would translate into greater relaxation and mental tranquility, which can facilitate falling asleep and improve its quality.

However, a study reported that it only found an improvement in sleep quality but without a significant effect on anxiety and depression (Fu et al., 2022). Similarly, another study found more substantial reductions in anxiety levels than in depression (Sun et al., 2022). It is important to note that the connections between stress, anxiety, depression, and sleep quality are clear and well-documented (Mayers et al., 2009; Blake et al., 2018; Kalmbach et al., 2018; Hertenstein et al., 2019). These conditions often coexist and influence each other in complex ways; for instance, not all people with insomnia will develop depression, and not all cases of depression are caused by insomnia (Turek, 2005). The interaction between these conditions is complex, and further research is needed to understand the underlying mechanisms and develop effective interventions.

The pandemic context adds another layer of complexity. Anxiety amplified by quarantine measures probably influenced the effectiveness of mindfulness in anxiety-related symptoms. In contrast, it did not have the same impact on depression, possibly because underlying factors that exacerbate depression, such as prolonged social isolation and hopelessness or experiencing multiple stressors, may have influenced the symptoms to decrease, although not substantially (Ettman et al., 2022).

Additionally, individual variability in mindfulness practice is a key factor that can influence intervention outcomes, especially in contexts where anxiety, depression, and sleep quality are sought. Some participants may be able to integrate mindfulness techniques more quickly, allowing them to experience immediate improvements in areas such as sleep quality and anxiety, which tend to respond promptly to emotional regulation and mental relaxation practices. However, participants with more entrenched depressive symptoms may have difficulty adopting these techniques in a short period as depression is linked to more chronic and complex thought patterns, which require a longer and more sustained approach (Breedvelt et al., 2019). Therefore, the duration of the intervention is another factor to consider, particularly when addressing chronic conditions like depression.

ANCOVA and the HC3 model showed substantial reductions in stress and anxiety, while depression exhibited more modest effects. These findings are consistent with a meta-analysis of mindfulness-based interventions in university students, where researchers reported significant reductions in stress and anxiety. However, the effects on depression were not as consistent; the evidence on sleep quality was inconclusive (Dawson et al., 2020). This suggests that while mindfulness has clear benefits for stress and anxiety, its limitations in treating depression warrant further investigation.

Our results also showed a significant improvement in social support and life satisfaction. Mindfulness training enhances the perception of social support and diminishes interpersonal sensitivity and negative emotions (Zhang, 2023). This may be because mindfulness fosters greater self-awareness and emotional regulation, helping people better manage their emotional reactions in social interactions (Freudenthaler et al., 2017). This allows individuals to be more empathetic and less reactive, improving the quality of their relationships and, therefore, the perception of social support (Acoba, 2024). Mindfulness practice also develops greater compassion toward oneself and others, encouraging more open and non-judgmental attitudes in social interactions (O’Connor et al., 2015; Godward et al., 2019).

This openness facilitates the construction of more substantial and genuine relationships, increasing the perception of social support. Mindfulness may enhance social awareness and relationship skills, which are essential components of social intelligence (Feuerborn and Gueldner, 2019) and are key to establishing good relationships in an academic context.

Besides, the mindfulness-to-meaning model indicates that mindfulness improves positive cognitive reappraisal and emotional regulation, leading to increased well-being (Garland et al., 2015). This improvement in self-perception and emotional regulation translates into greater life satisfaction. Evidence also suggests that both core self-evaluation and negative affect mediate the effect of trait mindfulness on life satisfaction, aligning with the mindfulness-to-meaning perspective. In particular, trait mindfulness influences life satisfaction through two mediation pathways— “core self-evaluation → positive affect” and “core self-evaluation → negative affect”—highlighting the combined significance of cognitive and emotional factors in understanding how trait mindfulness fosters life satisfaction (Li et al., 2022). These insights advance the theoretical understanding of how trait mindfulness relates to life satisfaction and offer valuable guidance for enhancing overall well-being.

In addition, ANCOVA and the HC3 model support these results, showing the most robust result for social support. This implies that mindfulness interventions help improve individual well-being and social dynamics, enhancing the sense of belonging to a community (Fagioli et al., 2023). Social support is a critical buffer against stress and promotes resilience, particularly in demanding settings such as university life or high-pressure workplaces (Hefner and Eisenberg, 2009; Feeney and Collins, 2015). By cultivating mindfulness skills, people may become more attuned to their social environments, foster more supportive connections, and have increased feelings of belonging and general well-being (Lindsay et al., 2019). This highlights the need to integrate mindfulness-based interventions into comprehensive strategies to improve social connectedness and mental health in educational settings. However, our results did not show a significant mediation effect of sleep quality, social support, or life satisfaction on anxiety, stress, or depression. Therefore, these variables are not mechanisms that explain the effect of mindfulness on stress and anxiety; instead, more specific mechanisms, such as rumination, worry, self-compassion, cognitive reactivity, aversion, attention regulation skills, and positive affect, could explain these effects (Maddock and Blair, 2023). However, in the case of depression with more established cognitive patterns, changes may be inconsistent depending on the severity and duration of symptoms (Diehr et al., 2006).

Regarding moderation, the results also showed no significant effects on sleep quality, social support, or life satisfaction as moderators in the relationships between mindfulness and anxiety, stress, or depression. A study found that factors such as the intensity and duration of stressors can affect the success of mindfulness in buffering stress or anxiety. However, these moderating effects depend on the specific types of stressors they face and how well individuals integrate mindfulness techniques into their daily lives (Westphal et al., 2021). In their study, these researchers also found that mindfulness moderated the adverse impact of low social support on depression, suggesting that individuals with higher levels of dispositional mindfulness were less affected by limited social support. At the same time, our study did not observe significant moderating effects of social support. This implies that the observed effects of the mindfulness intervention appear to be direct, at least in the context, population, and variables we studied, requiring further research on the possible mechanisms through which mindfulness impacts mental health.

Furthermore, a study emphasized the role of mindfulness in improving emotional regulation and reducing stress, suggesting that mindfulness facilitates better coping mechanisms by helping people decenter from negative emotions and reframe their experiences (Galindo et al., 2020). This resonates with the Mindfulness-to-Meaning Theory (Garland et al., 2015), which provides a theoretical framework that aligns with this study’s findings. According to this theory, mindfulness enables individuals to decenter from stress evaluations, fostering a metacognitive state that broadens attention to novel information. This process facilitates positive cognitive reappraisal, enhances emotional regulation, and fosters a sense of meaning in life. Therefore, mindfulness possesses the capacity to target core emotional and cognitive processes. These mechanisms may elucidate why mindfulness directly impacted stress, anxiety, and depression in this study without significant mediation through variables such as sleep quality or social support.

Cultural factors also play a role in mindfulness interventions among university students. Indeed, a Turkish version of an internet-based mindfulness intervention demonstrated feasibility and acceptability, although it showed limited improvements in depression and anxiety (Balci et al., 2024). In Indonesia, a culturally adapted internet-delivered mindfulness intervention significantly improved psychological distress and well-being among university students (Listiyandini et al., 2024). Besides, research on Asian American and European American college students found that acting with awareness and nonjudging were inversely associated with negative mental health outcomes in all groups, while observing was positively linked to anxiety and stress among Asian Americans (Jo and Pan, 2024). These findings underscore the importance of cultural and contextual factors in shaping mindfulness outcomes. The coastal Peruvian population studied here, distinct in its exposure to Westernized practices and stressors, likely influences the acceptance and effectiveness of mindfulness interventions. This may explain the moderate results observed for depression in this study, as well as the lack of significant mediation and moderation effects in the analyzed variables. However, Peru’s cultural diversity suggests that these findings might differ in highland or jungle populations, where traditional values and stressors are distinct. Moreover, these findings reinforce that mindfulness interventions may have direct effects in certain contexts, as observed here, rather than being mediated through variables such as social support or sleep quality. This direct effect could also be attributed to the unique socio-cultural dynamics of the coastal Peruvian population, suggesting that mindfulness practices must be adapted to resonate with specific cultural needs and expectations. By addressing these gaps, this research contributes to the growing body of evidence on mindfulness interventions’ global adaptability and limitations, particularly in underrepresented cultural contexts such as Peru. It also emphasizes the need for culturally tailored mindfulness practices to optimize outcomes in diverse populations.

The limitations of this study must also be considered. Although the sample size was adequate according to the power analysis, and the groups were homogeneous, coinciding with other similar studies in the Peruvian context (Alvarado García et al., 2018; Alvarado-García et al., 2022a, 2023a), a more significant number of participants could have increased the precision of the results, especially in moderation and mediation analysis. This would have allowed for greater generalization. Furthermore, the quasi-experimental design introduces a potential bias, limiting the ability to attribute the observed effects exclusively to the mindfulness intervention. The duration of the intervention, 12 sessions, may have been insufficient to observe significant improvements in some variables, such as depression, which may require more time to show changes. Likewise, the lack of long-term follow-up prevents the evaluation of the durability of the effects. In addition, only self-report instruments were used, which introduces the risk of response biases, such as social desirability bias or lack of precision in self-assessment. Physiological measures (such as heart rate variability or cortisol) were not included to complement self-reports and more objectively assess the effects of mindfulness on stress and anxiety. Another potential limitation is the selection of sections based on the willingness to participate, which can introduce a risk of selection bias. While the differences in willingness were not statistically significant, participants in these groups could have had higher initial motivation. This could influence their engagement with the intervention and the generalizability of the findings to less motivated populations. Future studies should consider randomized group assignments to mitigate this risk. In addition, future research may explore other moderating factors, such as resilience and family support. These limitations may guide future studies toward more robust designs with greater controls to generate a more complete understanding of the effects of the mindfulness intervention.

In addition, our findings underscore the practical value of incorporating mindfulness programs into university settings to reduce stress and anxiety and to enhance social support and life satisfaction. They also suggest that mindfulness primarily operates through emotional self-regulation and focused awareness mechanisms, as sleep quality and social support showed no significant mediating effects. This opens avenues for further research on cultural factors, the duration and intensity of mindfulness training, and other potential mediators—such as self-compassion and metacognition. Finally, the importance of adapting mindfulness interventions to specific sociocultural contexts, especially in regions with limited contemplative traditions, underscores the need for culturally sensitive approaches to optimize acceptance and impact.

## 5 Conclusion

The findings of this study indicate that the mindfulness intervention was effective in improving the mental health of university students, generating direct and significant effects on reducing stress, anxiety, and depression, as well as improving sleep quality, social support, and life satisfaction. The analyses performed, both ANCOVA and the robust HC3 model, support these findings, showing that the most robust improvements occurred in social support, highlighting the importance of this variable in general psychological well-being. However, no significant evidence of mediation or moderation by sleep quality, social support, or life satisfaction was found in the relationships between mindfulness and psychological variables, suggesting that the effects of mindfulness were direct in this context. Despite these limitations, the results support the implementation of mindfulness interventions to improve mental well-being in the university setting.

## Data Availability

The raw data supporting the conclusions of this article will be made available by the authors, without undue reservation.
