# Supplementary material for: Effect of a mindfulness program on stress, anxiety, depression, sleep quality, social support, and life satisfaction: a quasi-experimental study in college students
Source: Front Psychol. 2025 Feb 12;16:1508934. doi: 10.3389/fpsyg.2025.1508934 (PMC11864084; doi:10.3389/fpsyg.2025.1508934)
Supplement: Supplementary file 1 [file Table_1.docx]

**Supplementary Table S1**. **Descriptive and inferential statistics for stress, anxiety, depression, sleep quality, social support, and life satisfaction by group and study phases**

| **Groups** | Pretest | | Posttest | | p-Value^b^ |
| --- | --- | --- | --- | --- | --- |
|  | Median | IQR | Median | IQR |  |
| **CG** |  |  |  |  |  |
| Stress | 27.00 | (21.00-30.00) | 27.00 | (23.00-31.00) | 0.159 |
| Anxiety | 60.00 | (58.00-63.75) | 61.00 | (59.00-64.75) | 0.061 |
| Depression | 55.50 | (50.00-59.00) | 55.00 | (51.00-60.00) | 0.985 |
| Sleep Quality | 7.00 | (5.00-9.00) | 7.00 | (5.00-9.75) | 0.062 |
| Social support | 25.50 | (20.00-32.75) | 25.00 | (20.00-32.00) | 0.455 |
| Life satisfaction | 17.00 | (15.25-19.00) | 17.00 | (15.00-19.00) | 0.073 |
| **EG** |  |  |  |  |  |
| Stress | 26.00 | (22.75-29.00) | 19.00 | (15.00-21.00) | 0.000* |
| p-value^a^ | 0.770 |  | 0.000* |  |  |
| Anxiety | 59.50 | (57.25-63.00) | 51.00 | (48.00-55.75) | 0.000* |
| p-value^a^ | 0.716 |  | 0.000* |  |  |
| Depression | 55.00 | (50.00-61.00) | 50.00 | (48.00-55.00) | 0.000* |
| p-value^a.0^ | 0.694 |  | 0.000 |  |  |
| Sleep Quality | 7.00 | (4.00-8.75) | 4.00 | (2.00-6.00) | 0.000* |
| p-value^a^ | 0.825 |  | 0.000* |  |  |
| Social support | 30.00 | (21.25-33.00) | 55.00 | (46.00-61.00) | 0.000* |
| p-value^a^ | 0.201 |  | 0.000* |  |  |
| Life satisfaction | 18.00 | (14.25-19.00) | 25.00 | (22.00-29.00) | 0.000* |
| p-value^a^ | 0.810 |  | 0.000* |  |  |

*p<0.05

^a^p-value is calculated by the Whitney U test between groups.

^b^p-value is calculated by the Wilcoxon test between study phases.

This table presents the descriptive and inferential statistics for stress, anxiety, depression, sleep quality, social support, and life satisfaction. Median and interquartile range (IQR) values are shown for each group and phase. The Mann-Whitney U test was used for between-group comparisons, and the Wilcoxon test was used for between study phases comparisons. These data complement the main findings presented in the manuscript.
