# Supplementary material for: Effect of a mindfulness program on stress, anxiety, depression, sleep quality, social support, and life satisfaction: a quasi-experimental study in college students
Source: Front Psychol. 2025 Feb 12;16:1508934. doi: 10.3389/fpsyg.2025.1508934 (PMC11864084; doi:10.3389/fpsyg.2025.1508934)
Supplement: Supplementary file 2 [file Table_2.docx]

| **Sections** | **Willing to participate** | **Not willing to participate** | **Total** | **p-Value** |
| --- | --- | --- | --- | --- |
| **A** | 41 (61.2%) | 26 (38.8%) | 67(100%) | 0.938^a^ |
| **B** | 43 (63.2%) | 25 (36.8%) | 68(100%) |  |
| **C** | 41 (59.4%) | 28 (40.6%) | 69(100%) |  |
| **D** | 45 (64.3%) | 25 (35.7%) | 70(100%) |  |

**Supplementary Table S2. Participation Willingness Across Course Sections**

^a^p-value is calculated by the Pearson Chi-Square test

This table presents the preference survey results to assess students' willingness to participate in the mindfulness program. Data includes the number and percentage of students willing and not willing to participate from each course section (A, B, C, and D). The analysis shows no statistically significant differences in willingness to join between the sections (p = 0.938), as calculated using the Pearson Chi-Square test. These findings were used to guide the selection of sections for inclusion in the study.
